# Sleep and wake markers of thalamocortical functioning in early-course psychosis and first-degree relatives

**DOI:** 10.1038/s41537-026-00735-0

**Published:** 2026-03-11

**Authors:** Bengi Baran, Dan Denis, Dimitrios Mylonas, Hazal Arpaci, Courtney Spitzer, Nicholas Raymund, Christine Talbot, Erin Kohnke, Olivia Larson, Robert Stickgold, Matcheri Keshavan, Dara S. Manoach

**Affiliations:** 1 Department of Psychological and Brain Sciences, University of Iowa, Iowa City, IA USA; 2 Department of Psychiatry, Carver College of Medicine, University of Iowa, Iowa City, IA USA; 3 Iowa Neuroscience Institute, University of Iowa, Iowa City, IA USA; 4 Department of Psychology, University of York, York, UK; 5 Department of Psychiatry, Massachusetts General Hospital, Harvard Medical School, Boston, MA USA; 6 Department of Psychiatry, Beth Israel Deaconess Medical Center, Harvard Medical School, Boston, MA USA

**Keywords:** Psychosis, Biomarkers

## Abstract

Thalamocortical circuits regulate information flow between sensory inputs and higher-order processing, and their disruption is increasingly implicated in psychotic disorders. However, scalable biomarkers of this circuitry remain limited. We assessed P50 sensory gating, 40 Hz auditory steady-state responses (ASSRs) and sleep spindles in relation to resting-state thalamocortical connectivity in early-course psychosis (EC, *n* = 19), first-degree relatives (FHR, *n* = 24), and demographically matched non-psychiatric comparison subjects (NC, *n* = 28). Compared to NC, EC, and FHR exhibited hyperconnectivity of the thalamus with the primary auditory cortex. Patients showed spindle deficits and impaired sensory gating and ASSRs, while FHR showed abnormal ASSR. In the entire sample, sleep spindles and sensory gating were associated with distinct thalamic connectivity patterns involving sensorimotor and dorsolateral prefrontal cortices, respectively. Our multimodal, circuit-informed approach points to thalamocortical pathways as potential biomarkers of risk and targets for treatment in psychosis. These findings should be interpreted in light of the modest sample sizes and the cross-sectional design, and suggest that wake EEG measures, though scalable, may not fully capture sleep-related thalamic abnormalities.

## Introduction

Data from multiple sources, including genetics^1,2^, animal models^3,4^, human neuroimaging^5^ and sleep^6^ studies, implicate the thalamic reticular nucleus (TRN) and related thalamocortical circuitry in the etiopathology of schizophrenia (SZ). Neuroimaging studies reveal abnormalities in the structural and functional connectivity of thalamocortical networks in people with psychotic disorders^7–10^. These abnormalities are present before the onset of psychotic illness^11,12^ and predict conversion to psychosis among clinical high-risk samples^13^. These connectivity findings highlight the need to study the functional consequences of abnormal wiring of thalamocortical circuitry. Specifically, thalamocortical networks are involved in the generation of sleep spindles, oscillations characteristic of non-rapid eye movement (NREM) sleep that play a causal role in memory consolidation^14–17^. In people with chronic^18^ and early-course SZ^19^, sleep spindle deficits are associated with cognitive impairment. We have demonstrated that resting-state functional hyperconnectivity of the thalamus to somatosensory and motor cortex correlates with reduced sleep spindles in people with chronic SZ^20^. Sleep spindle generation requires TRN inhibition of thalamocortical neurons. Thus, impaired TRN inhibition may be the shared underlying cause of thalamic hyperconnectivity with sensorimotor cortex and reduced spindle activity. Sensory gating during wakefulness, the process that prevents higher-order cognitive function from being overwhelmed by repetitive or redundant stimuli, also requires TRN-mediated inhibition and is abnormal in SZ^21,22^. It remains unclear whether these putative endophenotypes of SZ, sleep spindles during NREM sleep and sensory gating during wakefulness, reflect shared or dissociable disruptions in thalamocortical pathways, particularly in individuals at familial risk for psychosis. The goal of the present work is to examine thalamocortical connectivity in early-course psychosis (EC) and first-degree relatives to identify trait-based alterations of the circuitry that are associated with sleep and wake-related electrophysiological biomarkers of SZ.

Sleep spindles are brief (~1 sec), 12–15 Hz waxing and waning oscillations that are characteristic of stages 2 (N2) and 3 (N3) NREM sleep. Sleep spindles are initiated through reciprocal interactions between GABAergic neurons of the TRN and thalamocortical relay cells and propagated by thalamocortical networks^23,24^. As reviewed extensively before^6,25^, spindle activity is reduced in chronic^18^ and early-course SZ^19^ as well as in those at familial^26–28^ or clinical high-risk for psychosis^29^. In a prior study, we investigated the relations of spindle deficits with thalamocortical communication in chronic SZ and NC subjects who completed a high-density EEG monitored sleep study to characterize NREM oscillations. In a separate session, they underwent MRI scanning, where we examined the resting-state functional connectivity of thalamocortical circuitry^20^. In line with previous work, we observed spindle deficits. We also observed increased thalamic connectivity with primary somatosensory and motor regions. Further, we demonstrated that abnormally increased connectivity of the thalamus with sensorimotor regions correlated with reduced sleep spindle density in both groups. This work identifies thalamocortical circuit dysfunction as a likely culprit in sleep spindle deficits in SZ.

During wakefulness, TRN regulates thalamocortical information processing by modulating the balance between externally driven sensory inputs and internally generated activity^30–34^. Sensory gating is thought to involve a broad neural network including the brainstem, thalamic circuits, hippocampus, and cortex^35^. Recent methodological advances in rodent studies have enabled precise monitoring and manipulation of TRN neurons, revealing their critical role in sensory gating: TRN neurons exhibit strong responses to initial auditory stimuli but reduced responses to repeated sounds^36^. Further, TRN activity correlates with improved selective attention^37^, and loss of TRN neurons impairs behaviors that require sensory selection^38^. Collectively, these findings support the thesis that thalamocortical circuitry, with the TRN as a key component, acts as a “gatekeeper” regulating sensory information flow to the cortex, leading to the study hypothesis that successful sensory gating would correlate with reduced resting-state functional connectivity of the thalamus with the sensorimotor cortex.

Sensory gating in humans is often quantified using the auditory evoked potentials P50 paradigm, which measures the amplitude of the P50 in response to a second auditory tone (S2; test stimulus) delivered shortly after an identical first tone (S1; conditioned stimulus). Deficits in sensory gating, indicated by elevated S2/S1 ratios, have been documented in psychotic disorders and in familial and clinical high-risk^22,39–43^, with these impairments correlating with cognitive deficits^44–46^ and symptom severity^47^.

Alongside P50 sensory gating, we incorporated the 40 Hz auditory steady-state response (ASSR) as a complementary wake EEG measure of excitation-inhibition balance relevant to thalamocortical circuit dysfunction in SZ. The ASSR reflects scalp EEG entrainment to 40 Hz click or noise trains, and is widely used as an electrophysiological proxy for gamma-band network synchrony. Gamma entrainment, like sleep spindles, is GABAergically mediated; it is thought to be generated by fast-spiking, parvalbumin-positive (PV + ) GABAergic interneurons in cortical and thalamic circuits^48–51^. TRN contains substantial PV+ neurons that are thought to be responsible for thalamocortical rhythm-generating activity^52^. Critically, gamma-band activity also plays a role in sensory gating. In awake primates, thalamocortical gamma-band activity selectively suppresses tactile inputs during passive limb movement^53^. Further, optogenetic work in rodents demonstrates that PV + TRN neurons preferentially drive spindle-like thalamocortical rhythms^54^. Although direct TRN contributions to 40 Hz ASSR remain less clearly established, the same study demonstrated that perturbing PV+ neuron activity in the cortex and basal forebrain alters sleep spindles and modulates 40 Hz ASSR power and phase consistency. This evidence links thalamocortical networks to ASSR generation and justifies including ASSR alongside P50 in the present study as wake EEG measures.

We examined whether sleep spindle impairments in psychosis reflect a common underlying thalamocortical dysfunction as wake EEG biomarkers. To this end, participants completed overnight sleep monitoring, wake EEGs, magnetic resonance imaging (MRI) scans, and clinical assessments over separate sessions. We tested people with early-course psychotic disorders, young first-degree relatives of people with SZ, and demographically matched NC subjects. By combining nocturnal EEG, wake EEG, and MRI in these samples, we aimed to identify multimodal biomarkers that would reflect trait-based alterations of thalamocortical circuitry associated with genetic risk for psychosis. We hypothesized that spindles, P50 sensory gating and ASSRs would be impaired in both psychosis and FHR. Further, we hypothesized that sensorimotor thalamocortical connectivity would be abnormally increased in both psychosis and FHR, and spindles and wake EEG measures would map onto the same sensorimotor thalamocortical networks in the entire sample. The measurement of sleep spindles requires nocturnal EEG and is prohibitively expensive and time-consuming for use in large-scale studies. Given the scalability challenges of nocturnal EEG, establishing concordance between sleep spindles and daytime EEG measures such as sensory gating and ASSR would support the use of the latter as accessible proxies of TRN circuit dysfunction in psychosis.

## Methods

### Participants

Sixty-seven individuals, ages 13–35, participated in the study. The sample consisted of EC, first-degree relatives of individuals with SZ who constituted the familial high-risk (FHR) group, and NC subjects. Groups did not differ in age, sex, body mass index (BMI) or parental education (Table 1). Study inclusion criteria are described in detail in Denis et al.^19^. Patients were recruited from Harvard-affiliated hospital outpatient programs, and diagnoses were confirmed by the Structured Clinical Interview for DSM-5 Axis I Disorders (SCID-5^55^). Individuals with no personal history of psychosis based on SCID or Schedule for Affective Disorders and Schizophrenia for School-Age Children-Present and Lifetime version (K-SADS-PL^56^) interviews, but a first-degree relative with SZ constituted the FHR group. Comparison subjects were recruited from the community and excluded for a personal history of mental illness confirmed by SCID, a family history of psychotic disorders, and treatment with medications known to affect sleep or cognition. All participants were excluded for substance use disorders in the past six months, unstable, non-psychiatric medical conditions that may affect sleep (e.g., diabetes, thyroid disease), pregnancy/breastfeeding; a history of head injury resulting in prolonged loss of consciousness (>15 min) or neurological sequelae; an intellectual disability, a diagnosed sleep disorder other than insomnia, neurological disorders, severe claustrophobia or metal in the body. The study was approved by the Mass General Brigham and Beth Israel-Deaconess Institutional Review Boards. After the procedures were explained, research staff obtained written informed consent from participants ≥18 yr, and consent of the parent and assent of participants <18 yr. All participants received monetary compensation.

**Table 1:** Participant characteristics.

	EC (*n* = 15)	FHR (*n* = 24)	NC (*n* = 28)	F(2,64)	*p*
Age	20.6 ± 3.8	22.9 ± 6.1	24 ± 4.5	2.31	0.11
Sex	7 F/8 M	17 F/7 M	12 F/16 M	*χ*^2^ = 4.5	0.11
BMI	26.9 ± 5.1	24.1 ± 4.1	24.7 ± 8.3	0.7	0.49
Estimated premorbid IQ^a^	96.6 ± 12.6	105.5 ± 14.9	100.4 ± 10.9	2.3	0.11
% Right-handed^b^	80%	59%	57%	*χ*^2^ = 2.4	0.30
Antipsychotic medication dosage^c^	170.3 ± 222.5	-	-		
Mean parental education	14.2 ± 3.8	15.5 ± 3.4	15.8 ± 2.9	1.1	0.35
MRI residual motion^d^	0.17 ± 0.06	0.16 ± 0.07	0.17 ± 0.13	0.1	0.90
MRI %Artifactual Slices	12.3 ± 7.4	10.7 ± 9.2	10.8 ± 7.9	0.2	0.81

^a^IQ (Intelligence Quotient), based on the Ammons Quick Test^60^.

^b^Based on the modified Edinburgh Handedness Inventory^88,89^ in which laterality scores of −100 and+100 denote exclusive use of left or right hand, respectively. Right-handedness was defined as scores ≥ 70.

^c^Calculated as chlorpromazine equivalents^75^.

^d^Root mean square of motion in the x, y and z directions after in-scanner prospective motion correction.

### Procedures

**Fig. 1:**
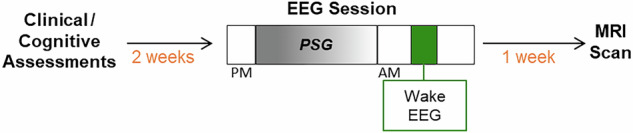
Study procedures relevant to the present study. This study utilized components from a larger study design aimed at investigating sleep-dependent memory^19^ and added wake electroencephalography (EEG) paradigms to their baseline sleep EEG monitoring session. An initial visit for clinical assessments, overnight EEG sessions and magnetic resonance imaging (MRI) scanning took place over a period of three weeks. The EEG session consisted of continuous sleep monitoring with polysomnography (PSG) and wake EEG paradigms for sensory gating and auditory steady-state responses in the morning.

Participants underwent clinical assessments, nocturnal EEG recordings, and MRI scans as part of a larger research protocol investigating NREM sleep oscillations and sleep-dependent memory consolidation^19^. The present study utilized this existing protocol and incorporated an additional wake EEG experiment to assess thalamocortical function during wakefulness (Fig. 1 depicts the protocol components relevant to the present study; the full study design is shown in Supplementary Fig. 1). Study procedures started with an office visit to obtain informed consent and to complete clinical and cognitive assessments. One week later, participants started the nocturnal sessions that included four nights of polysomnography (PSG) monitoring at the Massachusetts General Hospital or Beth Israel Deaconess Medical Center Clinical Research Centers. Each session consisted of 2 consecutive nights of PSG: the first night served as a baseline, and the second as an experimental night during which memory was tested. Participants engaged in their usual activities during the day. The following week, participants completed a second PSG session, in which the same procedure was repeated with a different memory task. The morning of the second baseline night, while still connected to the EEG acquisition devices, participants completed wake EEG paradigms to quantify sensory gating and ASSRs. Sleep data for the present study were obtained from this baseline night. Approximately one week after completing the EEG sessions, MRI scans were acquired for the characterization of thalamocortical functional connectivity.

### Cognitive assessments and clinical characterization

Psychotic symptoms were assessed with structured clinical interviews utilizing the Positive and Negative Syndrome Scale (PANSS^57^) in EC and FHR participants. The MATRICS Consensus Cognitive Battery^58,59^ was administered to all participants to assess: speed of processing, working memory, attention/vigilance, verbal and visual memory, reasoning and problem solving, and social cognition. Premorbid IQ was estimated with the Ammons Quick Test^60^; and current IQ was estimated with the Wechsler Abbreviated Scale of Intelligence(WASI-II^61^). Psychotic-like experiences (PLEs) in the entire sample were assessed using the Chapman Psychosis-Proneness Scales^62^, a self-report measure of subclinical schizotypal traits encompassing both positive (e.g., perceptual aberration, magical ideation) and negative (social anhedonia) dimensions.

### Sleep EEG acquisition and analysis

PSG data were acquired with either an Aura LTM64 (Grass Technologies, Astro-Med, Inc., RI) or an Embla RDx (Natus Medical Inc, CA) system, both with a sampling rate of 400 Hz. Sleep EEG data were not available from 2 participants (1 FHR and 1 NC) due to technical problems with recording. The electrode montage included 58 EEG electrodes on EasyCap EEG caps (Easycap GmbH, Herrsching, Germany), 2 submental electromyography, 2 electro-oculography and 2 mastoids positioned in accordance with the 10-20 system. Each 30-second epoch was visually scored as wake, N1, N2, N3, REM according to standard criteria^63^ by expert raters blinded to group. Artifacts were identified and removed automatically using Luna (https://zzz.bwh.harvard.edu/luna/). EEG data were preprocessed using custom MATLAB (MathWorks, Inc., Natick, MA) scripts and EEGLAB (https://sccn.ucsd.edu/eeglab/index.php) routines. Data were re-referenced to the average of the two mastoids, bandpass filtered between 0.3-35 Hz, and notch filtered at 60 Hz. Sleep spindles during N2 and N3 were automatically detected using a wavelet-based detector, validated for use in both non-psychiatric and SZ samples^18,64^. The raw EEG signal was subjected to a time-frequency decomposition using complex Morlet wavelets with a peak frequency of 13.5 Hz. Spindles were detected at each channel whenever the wavelet signal exceeded a threshold of 9 times the median signal amplitude of all artifact-free data for a minimum of 400 ms. This threshold is shown to maximize between class (“spindle” vs “non-spindle”) variance in previous samples of SZ and control patients^20,65^. We also extracted spindle amplitude, defined as the maximal voltage of a 4 sec window centered on the peak of the detected spindle.

ANCOVA models with the factor for Group (EC, FHR, NC) and a covariate for age were utilized to compare sleep spindles at each electrode. A nonparametric clustering method optimized for EEG^66^ was used to correct for multiple comparisons. Spatially adjacent electrodes with *p*_uncorrected_ < 0.05 were grouped into clusters based on the predefined neighborhood structure of the electrode array. Cluster-level statistics were calculated as the sum of F values within each cluster. Statistical significance was assessed by comparing the observed cluster statistic to a permutation-based null distribution of the maximum cluster statistic, generated by randomly permuting group labels 1000 times. Cluster-level *p* values reflect the probability of observing a cluster of that size or larger anywhere on the scalp under the null hypothesis. Clusters with corrected *p* < .05 (two-sided) were considered significant. To promote replicability and in accordance with recent EEG methodological guidelines^67^, we report the maximum effect observed within each cluster, quantified as partial eta squared (*η*_p_^2^).

### Wake EEG paradigms

EEG was recorded the morning of the PSG monitoring with the same acquisition system. Participants wore bilateral in-ear tubephones (Etymotic Research Inc., IL) and experimental stimuli were delivered with Presentation (Neurobehavioral Systems Inc., CA).

#### Sensory gating P50

The experiment consisted of EEG monitoring during the presentation of pairs of identical auditory stimuli. Each trial consisted of an initial tone that lasted 1 ms and a second identical tone that was separated by 500 ms. Trials were separated with an inter-trial interval of 10 seconds. The task consisted of 6 blocks of 40 trials each. Participants were instructed to sit still, keep their eyes open and study a map provided by the experimenter (to avoid falling asleep). The task lasted ~45 min.

Data were preprocessed and analyzed using custom scripts utilizing EEGLAB. EEG data from channel Cz were segmented into epochs starting from 100 ms before stimulus onset and ending 400 ms after stimulus presentation and filtered with a 10–50 Hz bandpass filter. Epochs with signal exceeding ± 100 μV were rejected as artifactual, there were no group differences in the number of trials rejected, F(2,54) = 0.23, *p* = 0.79. The remaining trials were averaged for each participant. The P50 was defined as the most prominent positive peak between 40 and 80 ms post stimulus onset for S1, and the preceding trough was used to calculate amplitude. The S2 peak was identified as the positive peak with a latency within 15 ms of that of S1. Two raters (BB and DD), blinded to group assignment, visually examined the averaged data for S1 for all participants. Based on consensus between raters, data from 10 participants (three NC and seven FHR) were excluded because of failure to detect a P50 peak for S1. Sensory gating was calculated as a ratio (S2/S1), a higher ratio reflects reduced gating. Group differences were tested using ANCOVA with age included as a covariate.

#### Auditory steady state responses (ASSR)

Participants were presented with a total of 150 trains of 1 ms white noise clicks delivered for a total of 500 ms with a 1100 ms stimulus onset asynchrony. There were a total of four blocks with stimulation frequencies of 20 Hz, 30 Hz, 40 Hz and 80 Hz. Participants were instructed to look at the fixation cross on the monitor throughout. The task lasted approximately 15 mins.

Data were preprocessed and analyzed using custom MATLAB scripts. ASSR data were not available for 13 participants (3 EC, 6 FHR and 4 NC) due to equipment failure or participant unavailability. Trials were epoched from −250 ms to 850 ms relative to stimulus onset. Epochs with signal exceeding ±100 μV were rejected as artifactual, there were no group differences in the number of trials rejected, *F*(2,54) = 0.54, *p* = 0.59. To correct for ocular artifacts, the epoched data were subjected to an independent component analysis (ICA), with ICA components reflecting eye blinks and movements being rejected. Clean, artifact-free trials were then subjected to a time-frequency decomposition using complex Morlet wavelets (7-cycle wavelet at 30 linearly spaced frequencies from 10 to 100 Hz), performed at electrode Cz. Average evoked power and phase consistency was then extracted for each stimulation frequency. Evoked power reflects phase-locked event-related changes in power relative to a 150–50 ms baseline period^68^, expressed in decibels (dB). Phase consistency reflects the variance of phases across single trials and ranges from 0 to 1, with a higher value indicating increased phase consistency. Group differences, controlling for age, were assessed using the same ANCOVA models and the cluster-based approach described for sleep oscillations, albeit with clustering over adjacent time-frequency points rather than electrodes.

### MRI data acquisition and analysis

Images were acquired using a 3T Siemens MAGNETOM Prisma scanner equipped with a 64-channel head coil and included a T1-weighted anatomical scan and two resting-state functional connectivity MRI scans, with parameters described in detail in the Supplementary Methods.

#### Preprocessing

Resting state functional scans were preprocessed using SPM8 (Wellcome Department, London, UK) implemented in MATLAB. Anatomical images were segmented into white matter, gray matter, and cerebrospinal fluid masks, corrected for slice acquisition time, spatially realigned to the reference image, resliced, and coregistered with the functional images. The volumes were normalized to the Montreal Neurological Institute (MNI) template and spatially smoothed using a Gaussian kernel with a full-width-at-half-maximum of 6 mm.

#### Quality assurance

Artifact Detection Tools (http://www.nitrc.org/projects/artifact_detect/) were used to identify and remove artifactual volumes (head displacement >1 mm from the previous frame, or the global mean intensity >3 SD above the entire functional scan). Residual head motion was calculated as the root-mean-square of translation parameters. There were no group differences in residual motion or the number of artifactual volumes (Table 1). No participants were excluded from MRI analyses.

#### Functional connectivity analyses

Subject-level analyses utilized the CONN Toolbox v17^69^ (implemented in Matlab), which uses the component-based noise reduction method, Anatomical CompCor^70^, rather than global signal regression, to remove physiological and other noise. Preprocessing involved applying a temporal bandpass filter of 0.008–0.09 Hz to the time series. Residual head motion parameters were added as regressors, and artifactual volumes were scrubbed in CONN. The thalamus was defined using the probabilistic FSL-Oxford-Thalamic-Connectivity-Atlas^71^ with a probability threshold of 25. Thalamocortical functional connectivity maps were generated for each participant by extracting the average time course of the BOLD signal from the bilateral thalamic seed and correlating it with every other gray matter voxel in the brain. The resulting Pearson coefficients were transformed into Fisher’s z. The connectivity analyses for the two runs for each subject were averaged.

Group differences in thalamocortical connectivity were assessed using voxel-wise ANCOVAs with group (EC, FHR, NC) as the between-subjects factor and age as a covariate. Pairwise post-hoc *t* tests were run when significant group effects were detected. We examined the relationships of sleep spindle density (in channel Cz) with thalamocortical connectivity using regression with group, spindle density, group-by-spindle density interaction and age as predictors, allowing estimation of the relations between spindles and functional connectivity while accounting for group-level differences. Whole-brain multiple-comparison correction used a cluster-based approach embedded in the CONN toolbox, applying a voxel-level uncorrected threshold of *p*_uncorrected_ ≤ 0.001 to define suprathreshold voxels and controlling false discovery rate (FDR) at the cluster-level (*p*_corrected_ ≤ 0.05). The same models were run, replacing spindle density with sensory gating ratios (in channel Cz) as well as ASSR evoked power and phase consistency (in channel Cz). Cz was selected as the standard midline electrode to maximize signal stability and comparability with prior literature.

#### Structural MRI analysis

Bilateral thalamic volume was estimated based on automated FreeSurfer subcortical segmentation^72,73^, which utilizes Bayesian inference to create a probabilistic atlas of 25 thalamic nuclei.

## Results

### Sleep EEG

Groups did not differ in sleep duration or architecture, with the exception of percentage of time spent in REM sleep, which was reduced in the FHR group in comparison to NC (*p* = 0.014) but not EC (*p* = 0.45) (Table 2).

**Table 2:** Group differences in clinical and cognitive assessments and sleep architecture.

	EC	FHR	NC	Stats	Post-hoc comparisons
PANSS^a^ total	55.3 ± 15	36.3 ± 6.2	NA	F(1,37) = 29.7, *p* < 0.001	
PANSS positive	12.3 ± 4.9	7.3 ± 0.63	NA	F(1,37) = 22.7, *p* < 0.001	
PANSS negative	15.4 ± 6	9 ± 2.6	NA	F(1,37) = 20.6, *p* < 0.001	
PANSS general	27.7 ± 6.7	19.9 ± 4.3	NA	F(1,37) = 18.6, *p* < 0.001	
Psychotic-like experiences^b^					
Magical ideation	11.7 ± 7.2	3.1 ± 1.8	2.2 ± 3.4	F(2,64) = 29.9, *p* < 0.001	EC > FHR = NC
Perceptual aberration	8.5 ± 8.7	3 ± 4.5	1.2 ± 2.7	F(2,64) = 10.4, *p* < 0.001	EC > FHR = NC
Social anhedonia	13.4 ± 8.8	7.4 ± 5.8	6.8 ± 5.7	F(2,64) = 6.3, *p* = 0.003	EC > FHR = NC
IQ (WAIS-II)^c^					
Verbal IQ	95.7 ± 14.1	110.4 ± 17.4	104.9 ± 12.1	F(2,64) = 5.1 *p* = 0.009	EC < FHR = NC
Performance IQ	101.8 ± 15.2	108.5 ± 16.5	106.7 ± 14.1	F(2,64) = 0.9 *p* = 0.38	
Full 4	98.4 ± 13.6	111.4 ± 15.1	106.6 ± 12.7	F(2,64) = 4.3 *p* = 0.018	EC < FHR = NC
MCCB^d^					
Speed of processing	41.6 ± 14.6	48.9 ± 13.3	50.9 ± 10.1	F(2,64) = 3.1, *p* = 0.054	
Attention	37.3 ± 14	47.9 ± 11.5	48.5 ± 9.8	F(2,64) = 5.9, *p* = 0.004	EC < FHR = NC
Working memory	46.4 ± 14.2	50.9 ± 11.7	53 ± 8.5	F(2,64) = 1.9, *p* = 0.16	
Verbal learning	44.3 ± 12.4	49 ± 10.9	50.8 ± 9.8	F(2,64) = 1.9, *p* = 0.16	
Visual learning	41.7 ± 12.7	46.2 ± 9.4	43.8 ± 9.5	F(2,64) = 0.7, *p* = 0.49	
Reasoning	46.2 ± 9.4	45.5 ± 11.2	46 ± 10	F(2,64) = 0.03, *p* = 0.97	
Social cognition	52.8 ± 15.5	56.5 ± 11.4	50.5 ± 8	F(2,64) = 1.6, *p* = 0.20	
Total composite score	39.4 ± 16	48.4 ± 12.8	48.2 ± 9.5	F(2,64) = 3.1, *p* = 0.053	
Self-reported sleep quality (PSQI^e^)	7.0 ± 4.6	6.2 ± 4.5	2.5 ± 2	F(2,64) = 9.6, *p* < 0.001	EC = FHR > NC
Sleep Architecture					
TST^f^	500.1 ± 58.9	476.2 ± 86.2	500.8 ± 84.5	F(2,62) = 0.71, *p* = 0.50	
SOL^g^	18.6 ± 24.2	28.2 ± 25.3	25.3 ± 17.2	F(2,62) = 0.78, *p* = 0.42	
WASO^h^	48.0 ± 33.7	62.1 ± 41.1	55.6 ± 45.7	F(2,62) = 0.53, *p* = 0.59	
% Sleep Efficiency	87.4 ± 6.7	82.7 ± 9.4	85.5 ± 8.1	F(2,62) = 1.58, *p* = 0.21	
%N1	5.7 ± 4.3	6.0 ± 3.9	5.8 ± 4.3	F(2,62) = 0.03, *p* = 0.97	
%N2	52.6 ± 11.2	54.0 ± 9.3	52.5 ± 7.4	F(2,62) = 0.20, *p* = 0.82	
%N3	23.2 ± 7.4	22.9 ± 9.6	20.3 ± 6.6	F(2,62) = 0.99, *p* = 0.41	
%REM	18.6 ± 7.0	17.1 ± 6.6	21.4 ± 4.7	F(2,62) = 3.33, *p* = 0.042	NC > FHR, NC = EC, EC = FHR

^a^PANSS: Positive and Negative Syndrome Scale^57^.

^b^Based on the Chapman Psychosis-Proneness Scales^62^.

^c^Wechsler Abbreviated Scale of Intelligence (WASI-II)^61^.

^d^MATRICS Consensus Cognitive Battery (MCCB)^58,59^.

^e^Pittsburg Sleep Quality Index (PSQI)^90^.

^f^TST Total Sleep Time duration in minutes.

^g^SOL Sleep Onset Latency duration in minutes.

^h^WASO Wake After Sleep Onset duration in minutes.

**Fig. 2:**
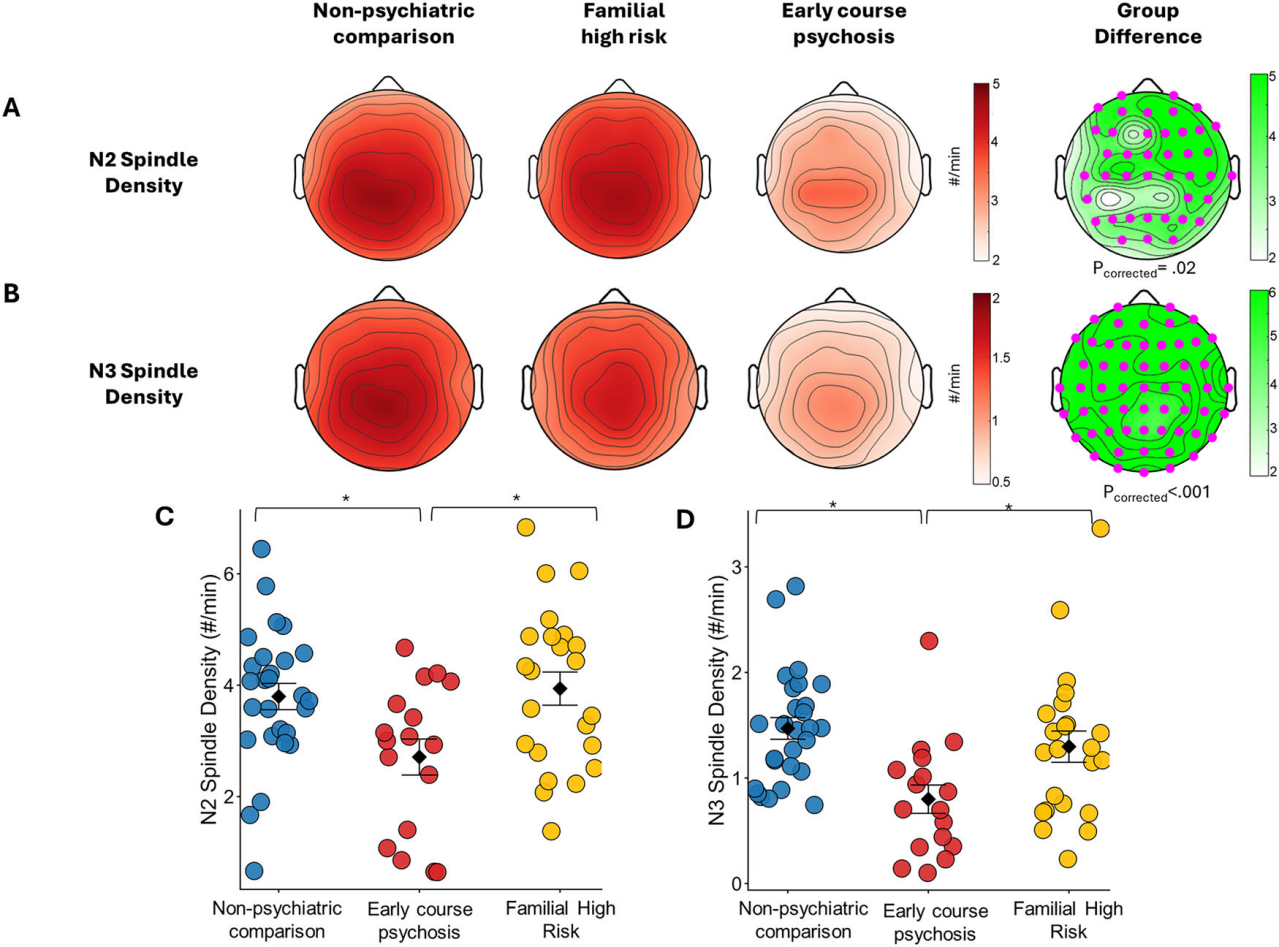
Sleep spindle density group differences. Topographical maps of sleep spindle density for **A** stage 2 non-rapid eye movement (N2) sleep and **B** N3 sleep. Topographical maps in the first three columns represent group means with warmer colors corresponding to higher values. The topographical maps in the fourth column represent F-statistics for the group differences. Electrodes in pink surpass cluster-level correction for multiple comparisons (brighter green corresponds to larger group effects). Dot plots of averaged sleep spindle density in the significant clusters for N2 (**C**) and N3 (**D**) spindles. Significant pairwise group differences are denoted with an asterisk.

We observed significant group differences in the density and amplitude of sleep spindles. Specifically, there was a significant main effect of Group for spindle density during N2 sleep (F_SUM_ = 175.33, *p*_corrected_ = 0.015, cluster size = 41 electrodes, Channel FC4 *η*_p_^2^ = 0.14 Fig. 2A, C), reflecting a reduction in spindle density in EC compared to NC (*p* = 0.01) and to FHR groups (*p* = 0.01) but no difference between NC and FHR (*p* = 0.71). This group difference was similarly significant for spindle density during N3 sleep (F_SUM_ = 409.4, *p*_corrected_ < 0.001, cluster size = all 58 electrodes, Channel T8 *η*_p_^2^ = 0.25 Fig. 2B, D), reflecting reduced spindle density in EC compared to NC (*p* < 0.001) and FHR (*p* = 0.01) but no difference between NC and FHR (*p* = 0.32). Sleep spindle amplitude for both N2 (F_SUM_ = 297.9, *p*_corrected_ = 0.003, cluster size = 54 electrodes, Channel FCz *η*_p_^2^ = 0.22) and N3 (F_SUM_ = 159.5, *p*_corrected_ = 0.022, cluster size = 38 electrodes, Channel FCz *η*_p_^2^ = 0.17) yielded similar group differences driven by deficits in the EC group (Supplementary Fig. 2). The effects of age were significant only for N3 spindle density (F_SUM_ = 129.4, *p*_corrected_ = 0.05, cluster size = 24 electrodes, Channel T8 *η*_p_^2^ = 0.095 Supplementary Fig. 3) corresponding to an age-related reduction in sleep spindle density.

### Wake EEG

#### Sensory gating P50

**Fig. 3:**
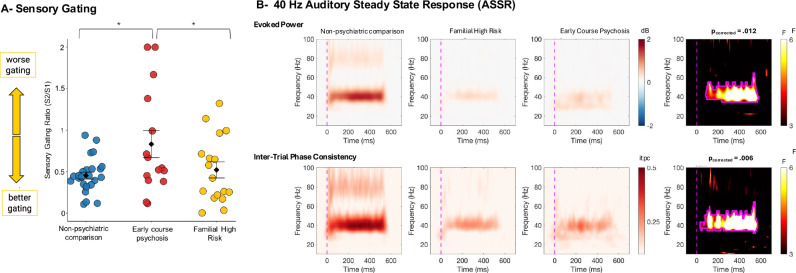
Wake EEG group differences. **A** Sensory gating ratios (S2/S1) at electrode Cz for each group. Higher values indicate worse gating. **B** Time-frequency maps of 40 Hz Auditory Steady State Response (ASSR) evoked power (top row) and inter-trial phase consistency (itpc; bottom row) at electrode Cz for each group, with warm colors representing higher and positive values (columns 1–3). The fourth column represents the F values for the group difference at each time-frequency point. Lighter colors represent stronger group differences (i.e. higher F values) for each time point. The clusters outlined in pink surpasses correction for multiple comparisons.

There was a significant main effect of Group (F(2,53) = 3.91, *p* = 0.026, *η*_p_^2^ = 0.13; Fig. 3A) corresponding to reduced gating (i.e. higher S2/S1 ratios) in EC compared to NC (*p* = 0.007) and to FHR (*p* = 0.038), but no differences between NC and FHR groups (*p* = 0.60). Neither age nor its interaction with Group predicted P50 sensory gating (*p*’s > 0.91).

#### Auditory steady state responses (ASSR)

Groups differed significantly for both metrics of the neural response to 40 Hz auditory stimulation, such that both early-course patients and relatives showed reduced power and phase consistency. Evoked power, after controlling for age, (Group main effect: F_SUM_ = 5221, *p*_corrected_ = 0.012, *η*_p_^2^ = 0.20, cluster size = 85–580 ms, Fig. 3B) was significantly reduced in EC compared to NC (*p* = 0.013) and in FHR compared to NC (*p* < 0.001) with no differences between EC and FHR (*p* = 0.49). Similarly, there was a significant main effect of group for the inter-trial phase consistency of the 40 Hz response (F_SUM_ = 6791, *p*_corrected_ = 0.011, *η*_p_^2^ = 0.24, cluster size = 33–568 ms, Fig. 3C), reflecting a significant reduction in phase consistency in EC compared to NC (*p* = 0.012) and FHR compared to NC (*p* < 0.001) but no difference between EC and FHR (*p* = 0.28). Age was not associated with either evoked power (*p*’s > 0.30) or inter-trial phase consistency (*p*’s > 0.37) of the 40 Hz response. There were no group differences in either the evoked power or the inter-trial phase consistency in response to 20 Hz, 30 Hz, or 80 Hz stimulation (see supplement for the statistics and Supplementary Figs. 4 and 5). Overall, relative to NC, both EC and FHR groups showed similar deficits in spectral power and phase locking for the 40 Hz auditory stimulation, with no differences observed at other stimulation frequencies.

### Thalamocortical connectivity

**Fig. 4:**
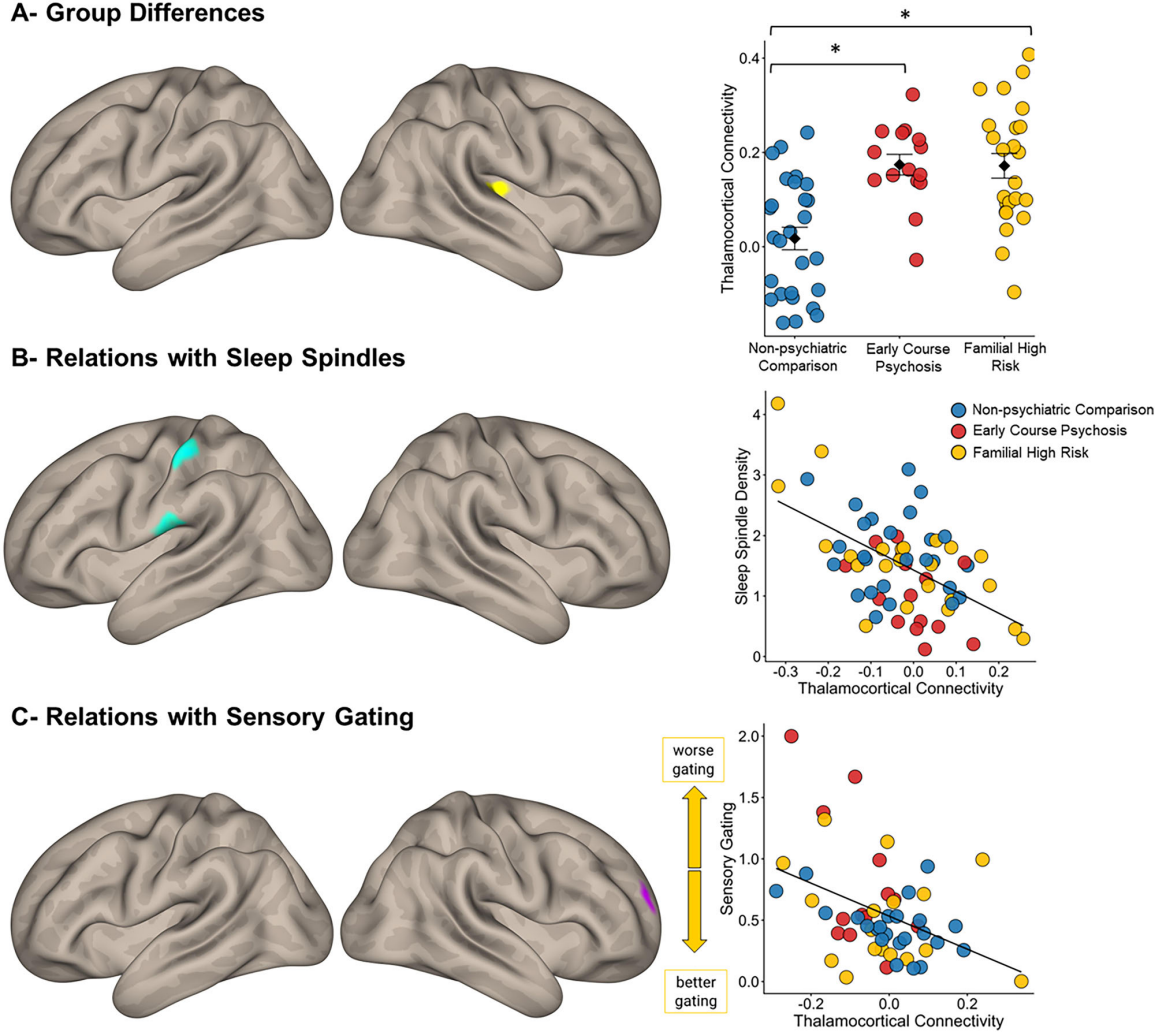
Thalamocortical connectivity analyses. In all panels, the right side shows the cortical clusters of significant thalamic connectivity on the cortical surface of the template brain (*p*_corrected_ ≤ 0.05) and the right side shows the dot plots of averaged thalamocortical connectivity in these clusters. blue: non-psychiatric comparison subjects; red: early-course psychosis; yellow: familial high risk. **A** Group differences in thalamocortical functional connectivity. Significant pairwise differences are denoted with an asterisk. **B** Relations of sleep spindle density with thalamocortical connectivity. The regression line is for the entire sample. **C** Relations of sensory gating with thalamocortical connectivity. The regression line is for the entire sample.

With respect to functional connectivity of the thalamus with every other gray matter voxel in the brain, the main effect of Group was significant in a cluster including portions of the right Heschl’s gyrus and insula (131 voxels, MNI peak: [40, −20,12], Fig. 4A, Table 3). This reflected that compared to NC, both the psychosis (t(38.02) = −4.90, *p* < 0.001, Cohen’s *d* = 1.41) and FHR (t(50) = −4.42, *p* < 0.001, Cohen’s *d* = 1.23) groups exhibited hyperconnectivity of the thalamus with the primary auditory cortex and related association areas, with no difference in thalamic connectivity between EC and FHR groups (t(36.81) = 0.07, *p* = 0.94; see Supplementary Fig. 6 for uncorrected group maps). The main effect of age was significant in two bilateral occipital clusters (MNI peak: [−38, −96, −4], 527 voxels and [34, −90, −2], 154 voxels, Supplementary Table 1 and Supplementary Fig. 7), reflecting an age-related increase in occipital thalamocortical connectivity in the entire sample.

**Table 3:** Maxima and locations of clusters showing significant effects in thalamic functional connectivity analyses.

Region	Voxels	MNI coordinates			BA	z-value (max)
		x	y	z		
Group differences						
R Insula	119	38	−22	12	13	14.3
Heschl’s gyrus		56	−22	10	41	
Relations with sleep spindle density						
L inferior parietal lobule	409	−48	−36	56	40	−4.1
Postcentral gyrus		−53	−18	55	2	
L Postcentral gyrus	168	−64	−20	20	40	−3.9
Relations with sensory gating						
R superior frontal gyrus	194	28	66	16	10	−4.9
R superior frontal gyrus		20	62	30	9	

All reported clusters have *p*_corrected_ ≤ 0.05 based on correction in the whole brain. Local maxima within the clusters (indented) are listed only if they fell in a different Brodmann area (BA) than the global maximum. *L* left, *R* right, *MNI* Montreal Neurological Institute

### Relations of sleep spindle density with thalamocortical connectivity

The whole brain regression model to predict thalamic connectivity relations with sleep spindles (in channel Cz), revealed a significant negative relationship between spindle density and thalamic connectivity with a cluster encompassing the left post-central gyrus (primary somatosensory cortex) extending to the left insula (Fig. 4B, Table 3). These relations did not differ by group, i.e. no significant spindle by group interaction. Analyses using N2 spindle density showed a similar pattern of inverse relationship with thalamocortical connectivity, but these effects did not survive whole-brain cluster-level correction (*p* = 0.087). Across the full sample, lower spindle density was associated with greater connectivity of the thalamus with primary somatosensory and interoceptive association areas of the cortex.

### Relations of wake EEG with thalamocortical connectivity

Thalamic connectivity analyses in relation to sensory gating revealed a different pattern. Sensory gating (P50 ratios in channel Cz) was inversely associated with thalamic connectivity with a cluster in the right middle and superior frontal gyrus, largely within the extended boundaries of the right dorsolateral prefrontal cortex (DLPFC; [28 66 16]; 194 voxels, BA 10; Fig. 4C, Table 3). These relations did not differ by group, i.e. no significant spindle by group interaction. This reflects that worse sensory gating in the entire sample was associated with reduced thalamic connectivity with the right DLPFC.

There was no overlap between the thalamocortical connectivity that predicts sensory gating and the thalamocortical connectivity that predicts sleep spindles. We did not observe any relations of ASSR (either evoked power or phase consistency in channel Cz) with thalamocortical connectivity.

### Thalamic volume

All thalamic volume analyses controlled for estimated total intracranial volume. We observed no group differences in volumetry for the whole bilateral thalamus (right: F(2,59) = 0.35, *p* = 0.71; left: F(2,59) = 0.27, *p* = 0.76). A separate model tested for group differences across anatomically defined thalamic nuclei (grouped as anterior, lateral, ventral, intralaminar, medial and pulvinar nuclei based on Thalhammer et al.^74^) separated by hemisphere and correcting for estimated total intracranial volume, which did not reveal a group difference (F(2,56) = 0.69, *p* = 0.51) or any interactions of group with nuclei or hemisphere (all *p*’s > 0.62). Thalamic volumetry was not associated with sleep spindles, sensory gating or ASSR metrics.

### Relations between sleep and wake EEGs and clinical variables

In the entire sample, lower spindle density was weakly associated with poorer P50 sensory gating, although this relationship did not reach significance (*r* = −0.24, *p* = 0.07). No other associations among spindle activity, sensory gating, or ASSRs were observed (all *p*’s > 0.15).

Finally, within the patient group, no significant associations were observed between clinical symptom ratings and spindle activity or wake EEG measures (all *p*s > 0.50). Across the full sample, self-reported psychotic-like experiences of magical ideation (Chapman Scales) showed moderate correlations with lower spindle density (*r* = −0.35, *p* = 0.004) and poorer sensory gating (*r* = 0.32, *p* = 0.014), but not with ASSR. Antipsychotic medication dosage in patients, measured as chlorpromazine equivalents^75^ was not associated with sleep spindle density, thalamocortical connectivity or ASSR measures (all *p*s > 0.65) but was significantly associated with P50 sensory gating, such that higher antipsychotic dose corresponded to worse gating (*r* = 0.77, *p* = 0.001).

## Discussion

We investigated the associations of sleep spindle activity and wake EEG biomarkers of psychotic disorders with thalamocortical functional connectivity in individuals with EC, first-degree relatives and NC subjects. Consistent with prior findings^9,76^, we observed abnormally increased thalamic connectivity with the auditory cortex in both psychosis and FHR groups. Sleep spindle and sensory gating (P50) deficits were specific to the psychosis group, while 40 Hz ASSRs were impaired in both patients and relatives. Importantly, thalamic connectivity was associated with both spindle activity and sensory gating, although the spatial distribution of these associations differed across measures. These findings extend our prior work linking sleep spindle abnormalities with thalamocortical dysfunction in chronic patients with SZ^20^ and provide new evidence that sensory gating impairments, a widely replicated biomarker of psychosis risk, also implicate thalamocortical functional connectivity. However, given the presence of sensory gating and spindle deficits only in patients and not in their first-degree relatives (in contrast to ASSR abnormalities which were also observed in relatives) our findings highlight heterogeneity in how different electrophysiological measures relate to thalamocortical circuitry across psychosis and familial risk and suggest that wake EEG measures may not fully substitute for sleep-based assessments as scalable proxies of thalamic dysfunction. Sleep spindle activity showed only a marginal and non-significant relationship with sensory gating, and no other sleep-wake EEG associations were observed. Taken together, these results suggest that sleep and wake-based EEG markers reflect complementary features of thalamocortical network function. Considering them jointly may provide a more complete characterization of circuit-level dysfunction across early psychosis and familial risk.

Thalamic connectivity with sensorimotor areas was inversely associated with spindle density, replicating our previous findings in chronic SZ and demographically matched comparison subjects^20^ and consistent with multiple studies demonstrating increased thalamocortical sensorimotor connectivity in both patients and unaffected first-degree relatives^5,7,9^. This finding lends support to our interpretation that thalamocortical hyperconnectivity reflects impaired TRN-mediated inhibition of thalamocortical relay activity. The observation that these associations extend to FHR individuals and comparison subjects is consistent with the hypothesis that spindle-related connectivity alterations are trait markers of vulnerability that appear on a spectrum, rather than state markers of psychotic illness. Moreover, the presence of thalamocortical connectivity abnormalities in minimally treated or antipsychotic-naïve patients suggests that these effects are unlikely to be solely attributable to medication effects.

We observed that impaired sensory gating is associated with reduced thalamic connectivity with a right prefrontal cluster in BA 9 and 10 that includes portions of the extended DLPFC^77^, with more efficient gating being associated with stronger thalamic-prefrontal functional coupling. This finding is consistent with studies in rodent models implicating the TRN in stimulus filtering and attention^37,38^, as well as human functional neuroimaging evidence showing that BOLD activation of the thalamus, hippocampus and DLPFC predicts sensory gating performance in SZ^78,79^ and EEG source-localization findings identifying the DLPFC and thalamus as joint generators of gating deficits in SZ^80^. Together, these results suggest a link between sensory gating and distributed thalamocortical dynamics and highlight the value of multimodal approaches to parsing circuit dysfunction. Although group-level differences in thalamic-prefrontal connectivity were not statistically significant in our sample, other imaging studies with larger samples (>200 participants) have revealed significantly reduced thalamic functional connectivity with prefrontal cortex in psychotic disorders as well as clinical high risk^9,13,81^. Nevertheless, our finding that individual variation in thalamic-prefrontal connectivity is associated with P50 ratios suggests that this circuit may contribute to individual differences in sensory gating. Because resting-state BOLD connectivity provides an indirect measure and has limited resolution for small thalamic structures, these thalamocortical connectivity findings should be interpreted cautiously. Further work using higher resolution human neuroimaging or causal approaches in animal models (e.g. optogenetic or chemogenetic methods targeting anatomically and functionally defined TRN subregions) will be necessary to clarify the underlying circuit mechanisms.

We did not observe any sleep spindle deficits in first-degree relatives (also see^19^), despite prior reports of reduced spindle activity in FHR groups^26–28^. Similarly, there were no sensory gating deficits in our FHR sample in contrast to previous work^43^. However, both ASSR deficits and increased thalamic connectivity with the auditory cortex were present in the first-degree relatives. Recent neuroimaging studies in FHR samples have also identified abnormalities in thalamic–auditory cortical circuitry, including Heschl’s gyrus and auditory association regions^82^. Of note, several meta-analytic studies of SZ endophenotypes in unaffected FHR samples report smaller effect sizes^83–85^ suggesting that shared genetic vulnerabilities or their phenotypic expression are highly heterogenous in unaffected first-degree relatives. In addition, FHR cohorts differ in developmental stage, clinical risk, and environmental exposures, factors that may influence whether electrophysiological or connectivity alterations are detectable. Speculatively, some relatives may be beyond the developmental period during which abnormalities are most evident, whereas others may exhibit compensatory or resilience mechanisms. Because relatives typically show attenuated deficits, larger samples are required to reliably detect group differences. Taken together, these unique properties of the FHR group could have limited our ability to detect more subtle abnormalities.

Several limitations of the present study should be acknowledged. First, the small sample size may have constrained our ability to detect small-to-moderate effects and limited the robustness of group comparisons. This was especially relevant for the FHR group, which includes individuals at varying levels of genetic risk for psychosis. The EC group was also modest in size, which limits our ability to distinguish SZ-specific effects from transdiagnostic features of psychosis. Within the patient group, none of the EEG or thalamocortical connectivity measures was significantly associated with symptom severity. Across the entire sample, psychotic-like experiences of magical ideation were moderately associated with spindle density and sensory gating. Additionally, data collection overlapped with the COVID-19 pandemic, which made recruitment challenging and may have introduced unmeasured stress-related or contextual confounds. Because EEG and fMRI were acquired on separate days, state-dependent variability may also have contributed noise to the associations observed. These factors highlight the need for replication in larger, longitudinal samples to further clarify the stability and specificity of thalamocortical biomarkers across the psychosis spectrum.

In contrast to sensory gating, ASSR measures were not significantly associated with thalamocortical connectivity. While prior work using magnetoencephalography and event-related fMRI indicates that 40 Hz ASSRs engage both the primary auditory cortex and subcortical auditory relay structures, including the thalamus^86^, their relationship to large-scale thalamocortical circuitry remains less well-characterised. One possibility is that ASSR reflects more localized cortico-cortical circuit abnormalities^87^ that are less affected by alterations in thalamocortical connectivity. Alternatively, the absence of significant associations could be due to insufficient sensitivity of resting-state fMRI to capture fast, stimulus-locked oscillatory dynamics that underlie the ASSR.

The present findings suggest that sleep spindles, P50 sensory gating, and ASSR serve as complementary indices of thalamocortical circuit function in psychosis and familial risk states. From a translational standpoint, these biomarkers may eventually help inform individualized risk calculation and early identification efforts for individuals at the greatest likelihood for progression to psychosis. Longitudinally, their sensitivity to thalamocortical dynamics suggests potential utility in tracking response to interventions. Moreover, our results raise the possibility that targeted strategies such as pharmacological agents or neuromodulation techniques that are aimed at enhancing spindle activity or strengthening thalamic-prefrontal coupling could be explored as potential avenues to address thalamocortical circuit deficits.

## Supplementary information

Revised Supplemental material - Marked up

## Data Availability

The data that support the findings of this study are available from the corresponding author upon reasonable request.
