# Supplementary material for: Sleep and wake markers of thalamocortical functioning in early-course psychosis and first-degree relatives
Source: Schizophrenia (Heidelb). 2026 Mar 11;12(1):40. doi: 10.1038/s41537-026-00735-0 (PMC13111589; doi:10.1038/s41537-026-00735-0)
Supplement: Supplementary file 1 — Revised Supplemental material - Marked up [file 41537_2026_735_MOESM1_ESM.pdf]

Supplementary Methods

MRI Scanner Parameters. Head stabilization was achieved with cushioning and participants wore earplugs (29dB rating) to attenuate noise. Automated shimming and alignment procedures were performed at the start of each scan[1]. Anatomical images were acquired using a 3D multiecho magnetization-prepared rf-spoiled rapid gradient-echo MEMPRAGE (T1 weighted) sequence with EPI based volumetric navigators for real time motion correction[2,3] (TR=2530 ms, Flip Angle=7°, TEs=1.69 ms/ 3.55 ms/ 5.41 ms/ 7.27 ms, GeneRalized Autocalibrating Partial Parallel Acquisition (GRAPPA) with an acceleration factor of two, FOV=256 mm; 176 in-plane sagittal slices; voxel size=1mm^3 isotropic; scan duration= 5m 34s). Two resting state functional connectivity MRI scans were obtained with a gradient-echo T2*-weighted sequence for blood oxygen level–dependent (BOLD) contrast (TR= 2000ms, flip angle=85°, TE=30ms, 32 contiguous horizontal slices parallel to the inter-commissural plane, voxel size=3.2mm^3 isotropic, interleaved, scan duration= 6m 6s). The functional sequences included prospective acquisition correction (PACE) for head motion[4].

Supplementary Results

No group differences for the Auditory Steady State (ASSR) neural responses for 20, 30 or 80 Hz stimulation survived correction for multiple comparisons (Supplementary Figures 3-4). Specifically, for stimulation at 20 Hz evoked power all $p_{corrected}$ >.80 and inter-trial phase consistency (itpc) all $p_{corrected}$ >.49; for stimulation at 30 Hz evoked power all $p_{corrected}$ >.41 and itpc all $p_{corrected}$ >.57; and for stimulation at 80 Hz evoked power all $p_{corrected}$ >.57 and itpc all $p_{corrected}$ >.66.

Supplementary Table 1. Maxima and locations of clusters showing significant age effects in thalamic functional connectivity analyses. All reported clusters have $p_{corrected} \leq .05$ based on correction in the whole brain. BA: Brodmann area; L: left; R: right, MNI: Montreal Neurological Institute.

Region	Voxels	MNI Coordinates x	y	z	BA	z-value (max)
Age effects						
L Inferior Occipital Gyrus	527	-38	-96	-4	18	5.55
R Inferior Occipital Gyrus	154	34	-90	-2	18	4.15

Supplementary Table 2. Summary of group differences for key electrophysiological measures and connectivity findings.

	EC	FHR	NC	Stats	Post-hoc comparisons
N2 Spindle Density	2.7 ± 1.3	3.9 ± 1.4	3.8 ±1.2	F_{SUM}= 175.33, $p_{corrected}$ = .015, η_p^2= .14	EC < FHR = NC
N3 Spindle Density	0.8 ± 0.6	1.3 ±0.7	1.5 ±0.5	F_{SUM}= 409.4, $p_{corrected}$ < .001, η_p^2= .25	EC < FHR = NC
N2 Spindle Amplitude	18.5± 5.6	23.1 ± 4.7	22.7 ±4.4	F_{SUM}= 297.9, $p_{corrected}$ = .003, η_p^2= .22	EC < FHR = NC
N3 Spindle Amplitude	17.4 ± 5.4	20.7 ± 3.9	20.7 ±3.9	F_{SUM}= 159.5, $p_{corrected}$ = .022, η_p^2= .17	EC < FHR = NC
P50 Sensory Gating	0.8±0.6	0.5±0.4	0.5±0.2	$F(2,53) = 3.91$, p = .026, η_p^2=.13	EC < FHR = NC
ASSR Evoked Power	.33±.36	.18±.28	.82±.79	F_{SUM}= 5221, $p_{corrected}$ = .012, η_p^2= .20	EC = FHR < NC
ASSR Intertrial Phase Consistency	.22±.11	.17±.09	.34±.15	F_{SUM}= 6791, $p_{corrected}$ = .011, η_p^2= .24	EC = FHR < NC
Thalamocortical Connectivity	.17±.08	.17±.12	.02±.12	131 voxels, MNI peak: [40,-20,12]	NC < EC = FHR

Supplementary Figure 1. The full study protocol and timeline. The current study utilized components from a larger project on sleep-dependent memory consolidation by adding a wake EEG experiment to one of the morning sessions. A baseline visit, two polysomnography (PSG) sessions and MRI scanning occurred across 21 days. Each PSG session consisted of two consecutive nights of PSG monitored sleep (baseline and memory nights). Wake EEGs were measured in the morning after the second baseline night. Sleep-dependent memory consolidation of a declarative and a procedural memory task was measured on the second PSG night of each session.

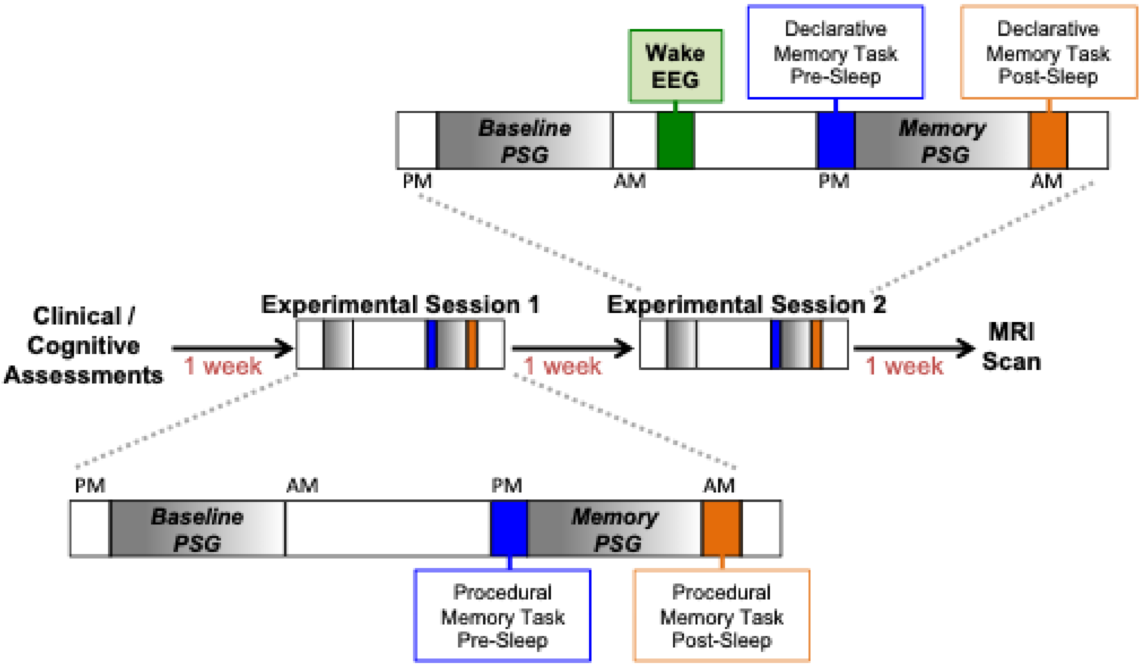

Supplementary Figure 2. Sleep spindle amplitude group differences. Topographical maps of sleep spindle amplitude, defined as the average values of maximal voltage of a 4 sec window centered on the peak of each of the detected spindle events during **(A)** stage 2 non-rapid eye movement (N2) sleep and **(B)** N3 sleep. For panels A and B, warmer colors on the first three columns represent higher spindle amplitude (in µV). Topographical maps on the fourth column represent F values for the group differences that account for age effects. Electrodes in pink surpass cluster-level correction for multiple comparisons. Dot plots of averaged sleep spindle amplitude in the significant clusters are shown in **(C)** for N2 and in **(D)** for N3 spindles. Significant pairwise group differences are denoted with an asterisk.

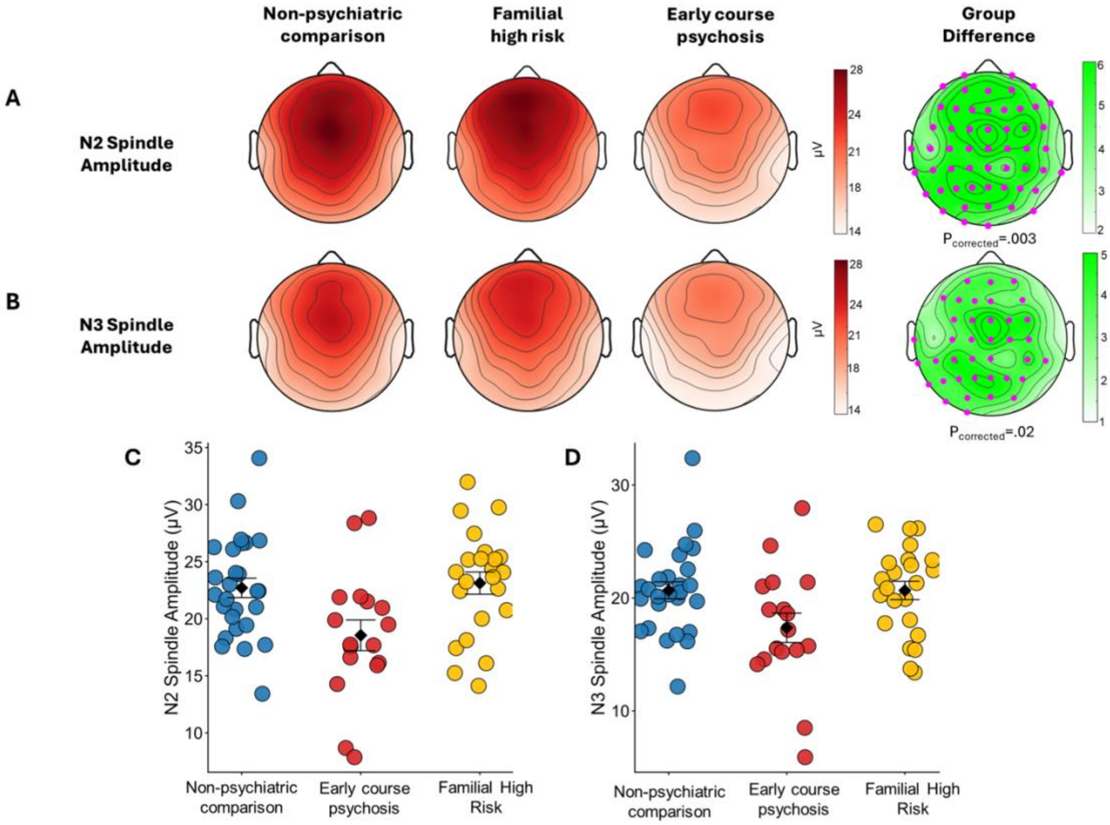

Supplementary Figure 3. Sleep spindle age effects. (A) Topographical maps of the F values for the main effect of age on N3 sleep spindle density. (B) Scatterplot of the relations between age and averaged sleep spindle density in the significant cluster. The regression line is for the entire sample.

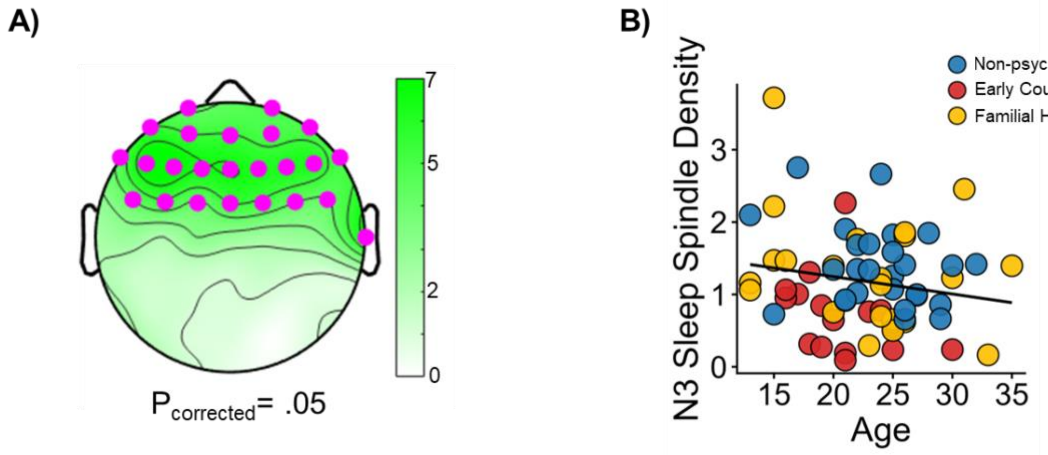

Supplementary Figure 4. Evoked power analyses for the Auditory Steady State Response. Time-frequency maps of ASSR evoked power responses at electrode Cz for each group (columns 1-3) for 20, 30, 40 and 80 Hz stimulation. Warm colors represent positive evoked power values. The fourth column represents the F values for the main effect of Group in ANCOVA models that controlled for age at each time frequency point. Lighter colors represent stronger group differences (i.e. higher F values) for each time point. For each time-frequency map, the x-axis indicates time (ms) and the y-axis indicates frequency (Hz). Group differences were significant (after correction for multiple comparisons) only for the 40 Hz stimulation.

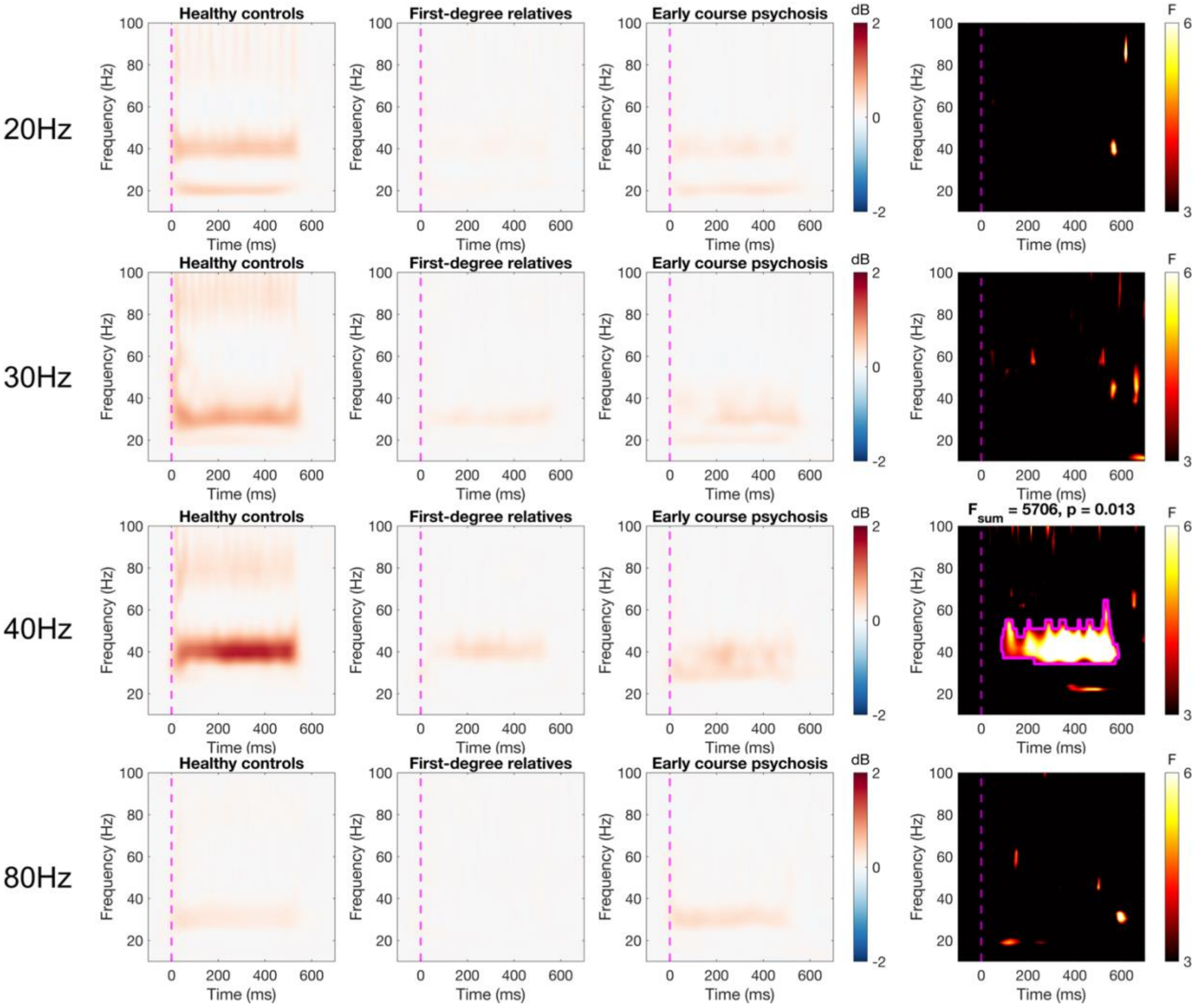

Supplementary Figure 5. Inter-trial phase consistency analyses for the Auditory Steady State Response. Time-frequency maps of ASSR inter-trial phase consistency (itpc) at electrode Cz for each group (columns 1-3) for 20, 30, 40 and 80 Hz stimulation. Warm colors represent higher itpc values. The fourth column represents the F values for the main effect of Group in ANCOVA models that controlled for age at each time frequency point. For each time-frequency map, the x-axis indicates time (ms) and the y-axis indicates frequency (Hz). Group differences were significant (after correction for multiple comparisons) only for the 40 Hz stimulation.

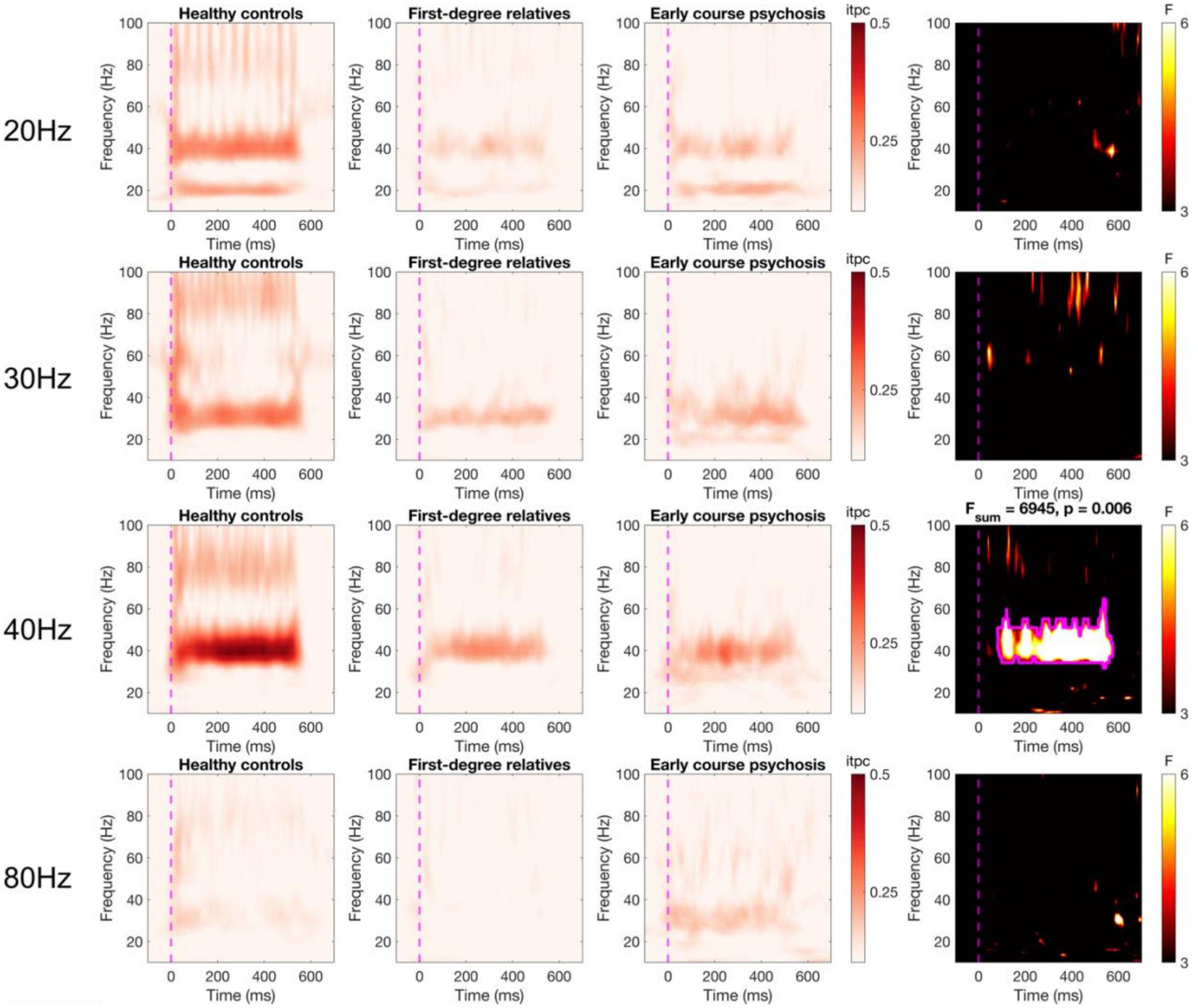

Supplementary Figure 6. Uncorrected thalamocortical connectivity maps. Unthresholded statistical maps of thalamocortical functional connectivity in (A) healthy controls and (B) schizophrenia patients displayed on the template brain. Positive connectivity is depicted in warm colors; negative connectivity is blue.

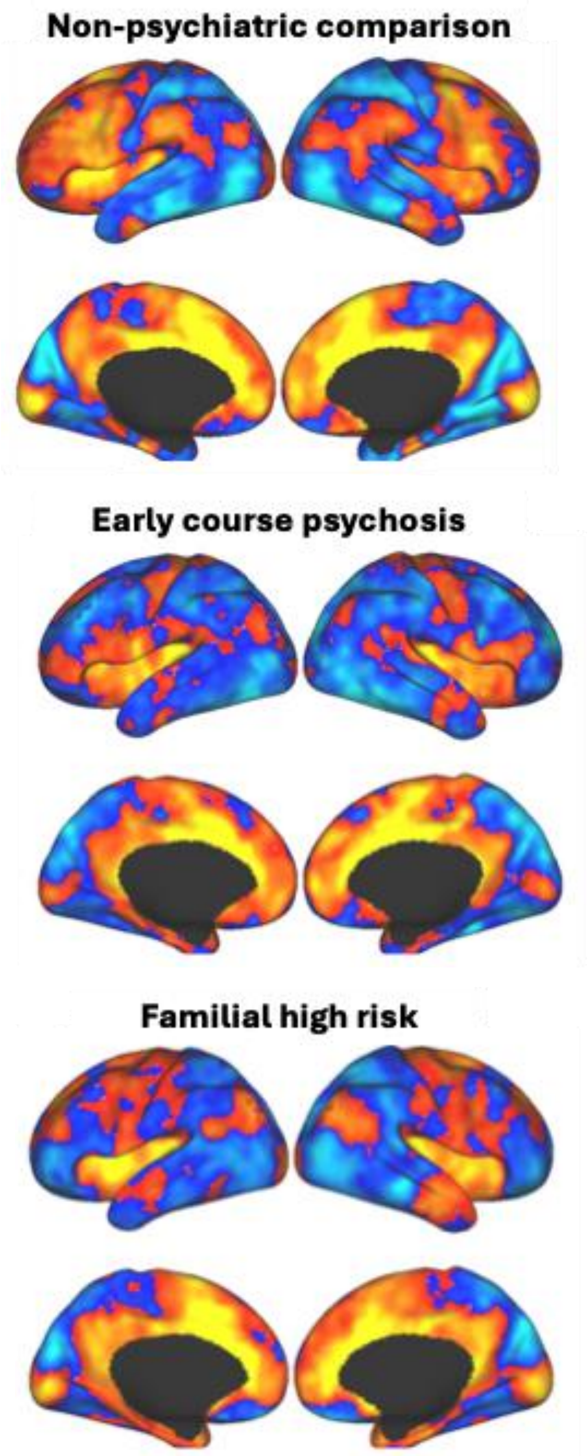

Supplementary Figure 7. Age effects for thalamocortical connectivity. The cortical clusters reflecting a significant main effect of age for thalamic connectivity displayed on the on the cortical surface of the template brain ($p_{corrected} \leq .05$).

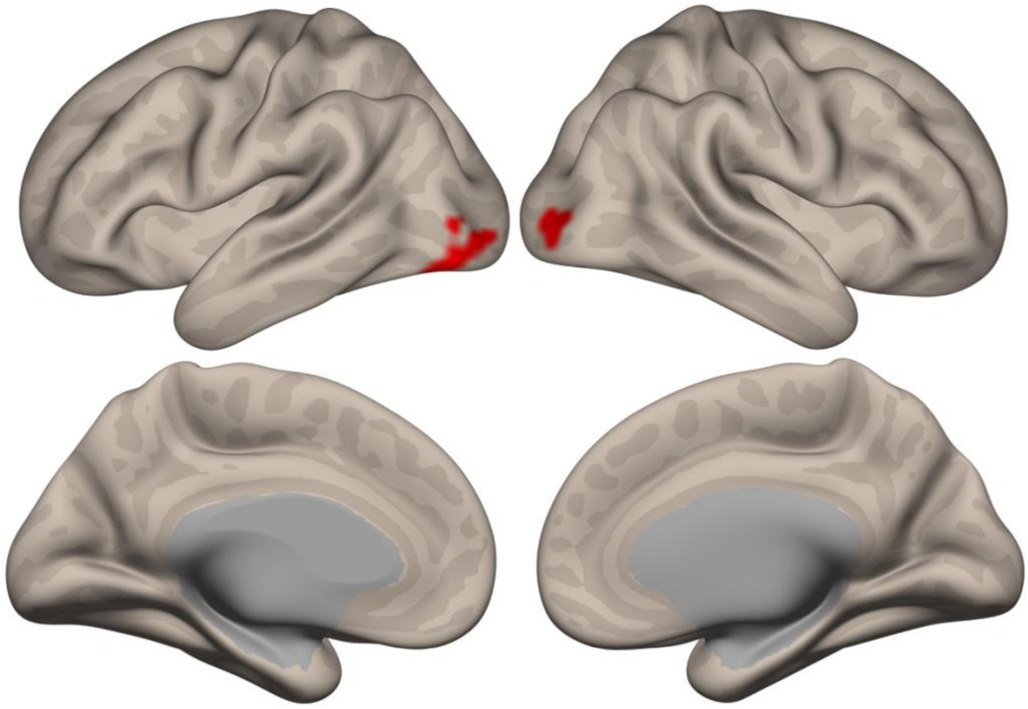

Supplementary References

1 van der Kouwe, A. J. W. *et al.* On-line automatic slice positioning for brain MR imaging. *NeuroImage* **27**, 222-230 (2005). https://doi.org/10.1016/j.neuroimage.2005.03.035

2 Tisdall, M. D. *et al.* Volumetric navigators for prospective motion correction and selective reacquisition in neuroanatomical MRI. *Magn Reson Med* **68**, 389-399 (2012). https://doi.org/10.1002/mrm.23228

3 van der Kouwe, A. J. W., Benner, T., Salat, D. H. & Fischl, B. Brain morphometry with multiecho MPRAGE. *NeuroImage* **40**, 559-569 (2008). https://doi.org/10.1016/j.neuroimage.2007.12.025

4 Thesen, S., Heid, O., Mueller, E. & Schad, L. R. Prospective acquisition correction for head motion with image-based tracking for real-time fMRI. *Magn Reson Med* **44**, 457-465 (2000).
